# Laparoscopic surgery assisted by colonoscopy for a submucosal cecal fecalith presenting as acute appendicitis: A case report

**DOI:** 10.1097/MD.0000000000008872

**Published:** 2017-11-27

**Authors:** Xiao-Jiao Ruan, Bai-Liang Ye, Zhi-Hai Zheng, Huan-Hao Zhou, Xiao-Feng Zheng, Zhen-Xu Zhou

**Affiliations:** Department of General Surgery, the First Affiliated Hospital of Wenzhou Medical University, Wenzhou City, Zhejiang Province, China.

**Keywords:** appendicitis, cecum, fecalith, submucosal lesion

## Abstract

**Rationale:**

A cecal submucosal fecalith is extremely rare and is likely to be misdiagnosed as appendicitis with an incarcerated fecalith.

**Patient concerns:**

This review presents the case of a female patient complaining of recurrent abdominal pain in the right lower quadrant, similar to the clinical symptoms of appendicitis. Physical examination revealed an abdominal tenderness in the right lower quadrant without rebound tenderness or muscular tension. An ultrasound examination found a mass located in the right lower abdomen. Computed tomography showed a high-density shadow in the cecal cavity.

**Diagnoses:**

A fecalith was detected in the submucosal cecal wall. The postoperative pathologic examination showed that the fecalith was located in the submucosa.

**Interventions:**

A partial cecal excision was performed under laparoscopic surgery assisted by colonoscopy.

**Outcomes:**

The patient was discharged 1 week after surgery without postoperative complications.

**Lessons:**

Fecaliths should be considered in the differential diagnosis of submucosal occupying lesions of the cecum.

## 1 Introduction

Cecal fecaliths are common in the cecal cavity, but are extremely rare as a cecal submucosal lesion. Preoperative diagnosis is difficult and the clinical symptoms are similar to appendicitis. Radiological imaging may show a high-density shadow in the cecum, which is likely to be misdiagnosed as appendicitis with an incarcerated fecalith. Enteroscopy shows a bulging mass on the cecal cavity wall without a mucosal ulcer, which could be misdiagnosed as a submucosal neoplasm. As reported, a cecal submucosal fecalithis a symptomatic or presents as intestinal hemorrhage^[1,2]^; however, a cecal submucosal fecalith leading to recurrent right lower quadrant abdominal pain, similar to the clinical symptoms of appendicitis, has not been reported. Herein, we report a female patient presenting with recurrent right lower quadrant abdominal pain, which was first misdiagnosed as appendicitis.

## 2 Case report

The report was approved by Institutional Review Board of The First Hospital of Wenzhou Medical University, and written informed consent was provided by the patient. A 65-year-old woman complained of a recurrence of right lower quadrant abdominal pain over the previous 10 years. Upon admission, she complained of exacerbated pain over the preceding week. She reported no fever or rigors. She denied any history of a recent altered bowel habit or urinary disease. The vital signs were normal. Physical examination revealed an abdominal tenderness in the right lower quadrant without rebound tenderness or muscular tension. No mass was palpated in the abdomen. In addition, the obturator sign, the psoas sign, and the Rovsing sign were all negative.

Blood routine examination was normal except for a C-reactive protein of 53 mg/L. Other tests, including measurement of the levels of electrolytes, urea, and amylase, as well as liver function test, were all normal. The levels of carcinoembryonic antigen, alpha-fetoprotein, and carbohydrate antigens were 1.1 ng/mL, 2.75 ng/mL, and 7.0 U/mL, respectively.

**Figure 1:**
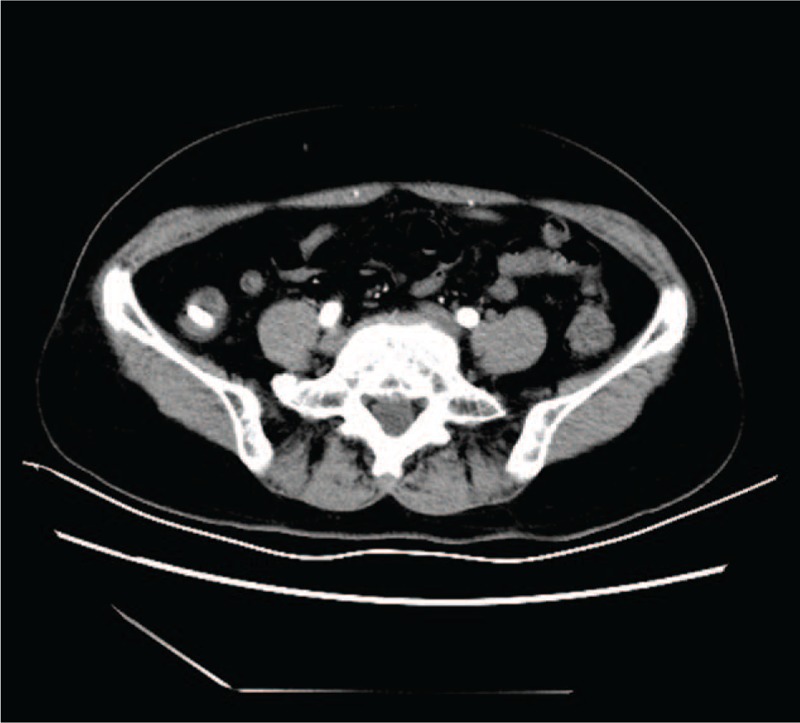
Computed tomography (CT) scan showing a high-density nodular shadow in the cecal cavity, approximately 0.8 cm×1.5 cm in size.

An ultrasound examination revealed a mass located in the right lower abdomen. A computed tomography (CT) scan showed a high-density shadow in the cecal cavity (Fig. 1).

**Figure 2:**
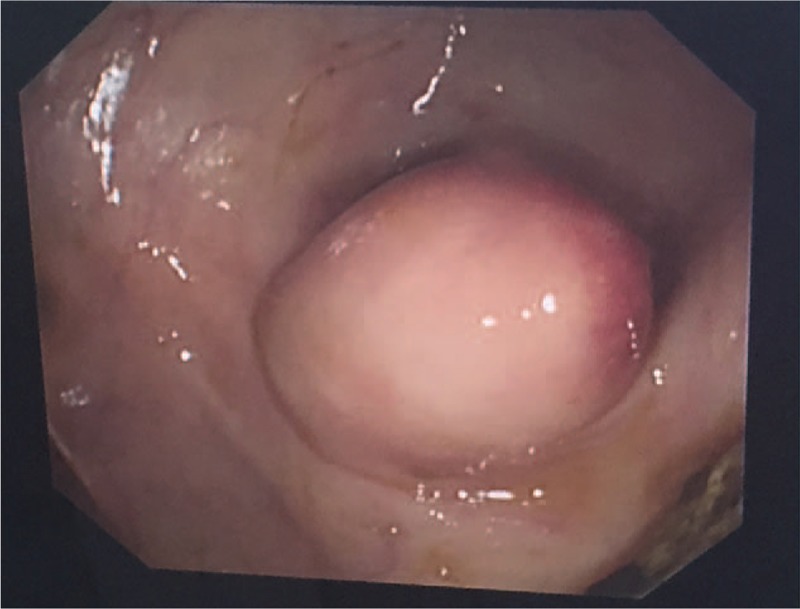
Enteroscopy showing a submucosal bulging lesion within the cecal cavity without a mucosal ulcer, approximately 1 cm×1.5 cm in size.

**Figure 3:**
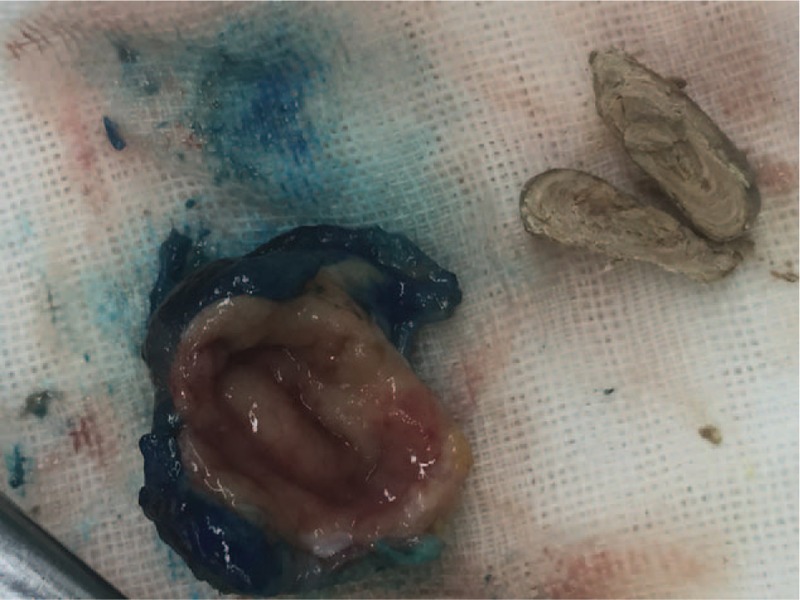
A bulging mass can be seen in the mucosa of the cecal wall. A portion of the fecalith, approximately 0.8 cm × 1.5 cm in size, was detected within the mass.

Our suspected diagnosis was appendicitis caused by an incarcerated fecalith. The pain was mitigated by antiinflammatory therapy with cefoperazone sulbactam sodium. A laparoscopic exploration was performed under general anesthesia. During the operation, the appendix was found to be of normal size with no evidence of acute inflammation. No mass or other abnormal lesion was encountered during exploration of the serous surface of the cecum or the ascending colon. Appendectomy was performed, but no fecalith was detected in the appendicular cavity. A submucosal bulging lesion without a mucosal ulcer in the wall of the cecum was detected by intraoperative enteroscopy (Fig. 2). The bulging lesion was marked with methylthioninium chloride and a needle. Finally, a partial cecal excision was performed under direct enteroscopic vision, and the mass was found to be a submucosal fecalith (Fig. 3).

**Figure 4:**
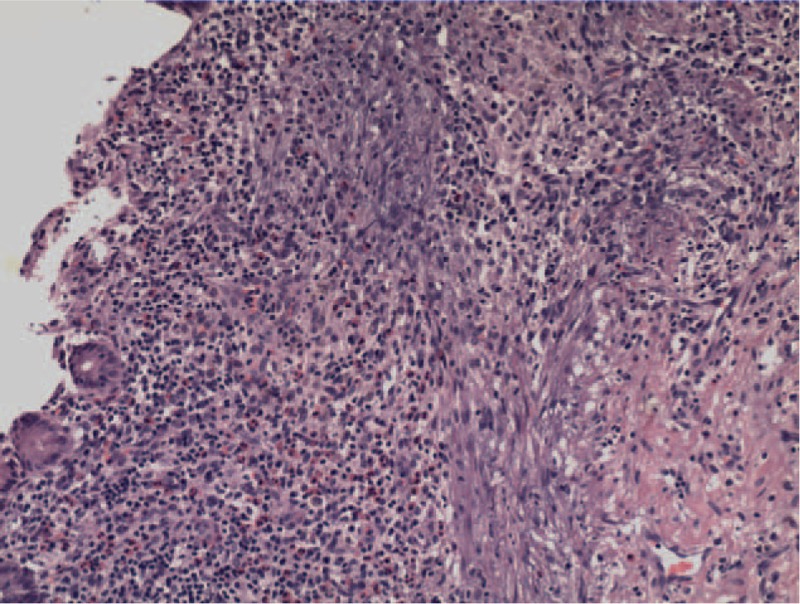
Different types of chronic inflammation and cellular infiltration, including lymphocytes, plasma cells, neutrophilic leukocytes, and eosinophils, were detected in the mucous membrane between the fecalith and intestinal muscular layer. This was accompanied by inflammatory exudation and erosion of the mucous membrane.

The pathologic examination showed that the cecal wall layer was intact and a fecalith was detected, which was the possible cause of the submucosal inflammation (Fig. 4). The patient was discharged 1 week after surgery without postoperative complications.

## 3 Discussion

A variety of lesions have been reported presenting as submucosal lesions in the cecum, most commonly including lipomas, gastrointestinal stromal tumors, and appendicular mucoceles.^[3–5]^ A submucosal fecalith presenting as a tumor-like bulging lesion has been rarely reported in clinical settings. It was difficult to distinguish whether the fecalith was located in the appendicular cavity or cecal diverticulum, which may be misdiagnosed as appendicitis or diverticulitis with an incarcerated fecalith. The formation mechanism of a submucosal fecalith remains unclear, and the possible explanation may be the result of a long-term incarceration of an appendicular fecalith. A submucosal cecal fecalith may induce right lower quadrant abdominal pain that is similar to appendicitis or may present as intestinal hemorrhage.

Shadi reported 1 cecal submucosal lesion detected during enteroscopy because of ulcerative colitis, and the submucosal lesion had an intact mucous membrane. Subsequently, a laparoscopic-assisted right partial colectomy was performed, and a fecalith was found near the appendicular orifice.^[1]^ Matthew described an intestinal hemorrhage induced by a cecal fecalith, which was managed by cecal resection; the fecalith was located proximal to the ileocecal valve and was extruded from the cecalcavity.^[2]^

Until recently, the diagnosis and treatment of cecal fecaliths have remained poor. In this report, a preoperative CT scan demonstrated a high-density shadow and indicated the presence of a fecalith. Based on the symptom similarities and imaging findings, the most common initial diagnosis is appendicitis or diverticulitis with an incarcerated fecalith.

The fecalith was most likely trapped in the appendicular orifice and, with time, resulted in the appearance of the presumed submucosal occupying lesion. The etiology of the lesion was not clear despite a CT scan and endoscopic ultrasonography. Fecaliths should be considered in the differential diagnosis of submucosal occupying lesions of the cecum.
